# Modeling Kick-Kill Strategies toward HIV Cure

**DOI:** 10.3389/fimmu.2017.00995

**Published:** 2017-08-28

**Authors:** Esteban A. Hernandez-Vargas

**Affiliations:** ^1^Frankfurt Institute for Advanced Studies, Frankfurt am Main, Germany

**Keywords:** HIV infection, HIV cure, reservoirs, mathematical modeling, LRA, ART, vaccination

## Abstract

Although combinatorial antiretroviral therapy (cART) potently suppresses the virus, a sterile or functional cure still remains one of the greatest therapeutic challenges worldwide. Reservoirs are infected cells that can maintain HIV persistence for several years in patients with optimal cART, which is a leading obstacle to eradicate the virus. Despite the significant progress that has been made in our understanding of the diversity of cells that promote HIV persistence, many aspects that are critical to the development of effective therapeutic approaches able to purge the latent CD4+ T cell reservoir are poorly understood. Simultaneous purging strategies known as “kick-kill” have been pointed out as promising therapeutic approaches to eliminate the viral reservoir. However, long-term outcomes of purging strategies as well as the effect on the HIV reservoir are still largely fragmented. In this context, mathematical modeling can provide a rationale not only to evaluate the impact on the HIV reservoir but also to facilitate the formulation of hypotheses about potential therapeutic strategies. This review aims to discuss briefly the most recent mathematical modeling contributions, harnessing our knowledge toward the uncharted territory of HIV eradication. In addition, problems associated with current models are discussed, in particular, mathematical models consider only T cell responses but HIV control may also depend on other cell responses as well as chemokines and cytokines dynamics.

## 1 Introduction

According to UNAIDS estimates for the year 2015, 36 million persons are infected with the HIV worldwide, and there are approximately 2.3 million new infections and 1.6 million AIDS-related deaths that occurred that year (1). Combined antiretroviral therapies (cART) are not able to eradicate the virus and HIV rebounds if therapy is discontinued. Upon HIV infection, a subset of latently infected cells carrying transcriptionally inactive integrated proviral DNA (the HIV reservoir) is rapidly established (2, 3). These cells are the main force behind HIV persistence under cART and, therefore, the main obstacle for an HIV cure (4–6). Thus far, there is only one reported case of a potential cure, known in the popular press as the Berlin patient (7). Unfortunately, the unique circumstances of the Berlin patient case would make it highly implausible to achieve a cure on large scales (8).

**Figure 1:**
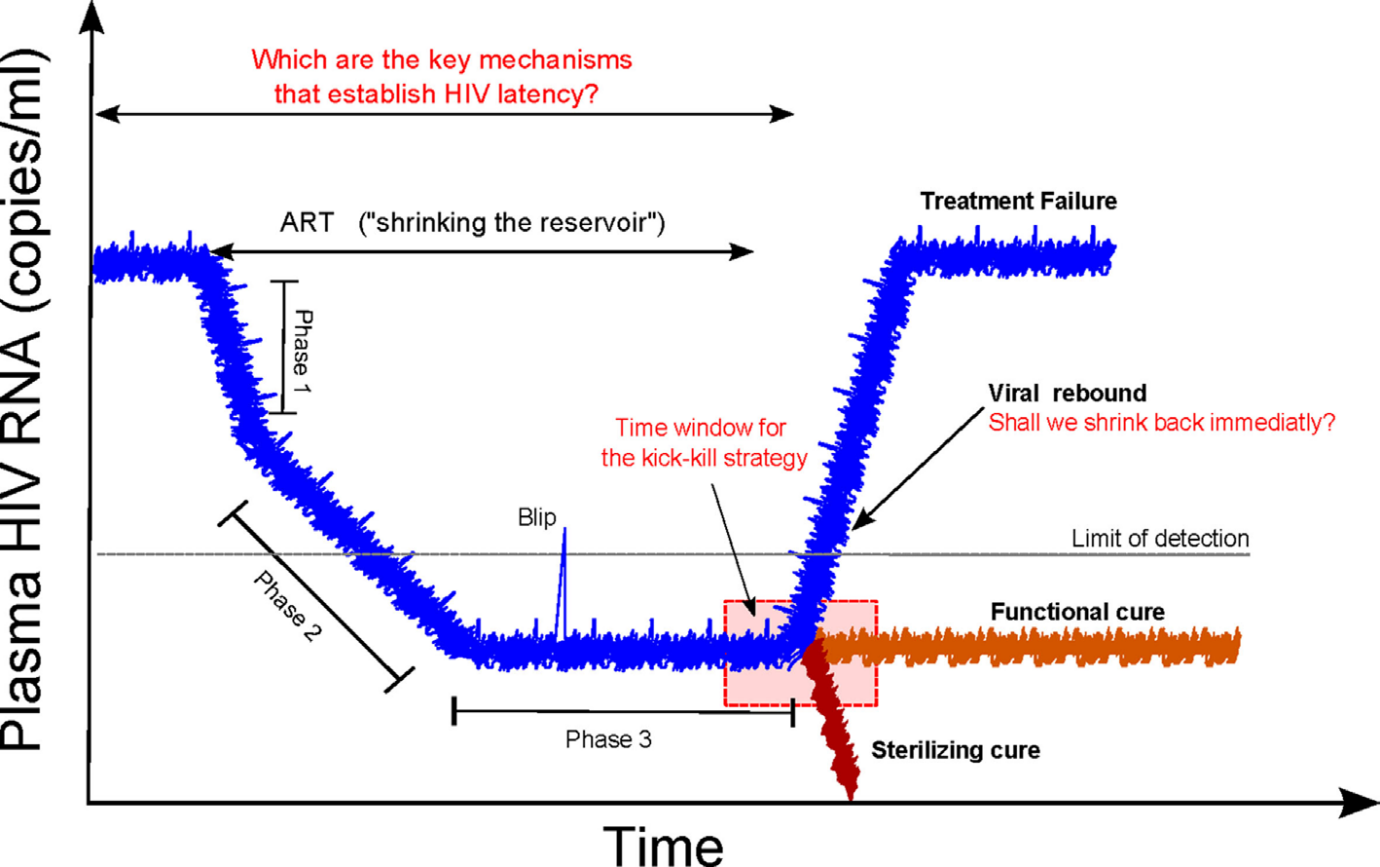
Shrink-kick-kill strategies toward an HIV cure. HIV undergoes three phases after cART initiation. The first phase describes the rapid decay of productively infected cells, e.g., activated CD4+ T cells. The second phase is led by cells that possess a half-life of about 14 days, which are not completely identified but are possibly macrophages and dendritic cells. The third phase is a low but stable level of residual viremia giving a plateau phase, which contains occasional viremic episodes (called blips). This third phase has been attributed to long-term reservoirs maintained by activation of latently infected memory CD4+ T cells. Recently, it has been hypothesized that tailoring a kick-kill strategy after cART cessation could lead to a sterilizing cure or a functional cure, i.e., achieving a controlled viremia below detection. This figure is a modification from Ref. (11).

Two different approaches are envisaged for curing HIV infection: a sterilizing cure if there is a complete eradication of the virus and infected cells; and a functional cure if there is permanent control of viral replication without therapy (2). There was a growing recognition that a cure for HIV infection could be feasible (4, 8, 9). Recent clinical observations have hypothesized that an early initiation of cART is crucial to a progressive contraction of the latent HIV reservoir (“shrink”). This could possibly be accomplished with simultaneous strategies that activate (“kick” or “shock”) the latent reservoir and increase the clearance of virus-infected cells (“kill”), known as a “kick-kill” or “shock-kill” strategy (10, 11). The time window to intervene at an early stage of infection, while reservoirs are limited, is envisaged to be narrow but critical to the performance of an effective “shrink-kick-kill” strategy (Figure 1).

Disentangling the leading mechanisms of HIV reservoirs is essential for the design of optimal therapeutic strategies. Although there are animal models such as the non-human primate and the BLT humanized mouse available to recapitulate HIV infection or even eradication, they are not perfect (12). Mathematical models can serve as a framework to interpret data of ongoing clinical trials, to evaluate the long-term of new therapeutic interventions, and to tailor future clinical trials. HIV modeling research twisted in a new dimension when the two works from Perelson et al. (13) and Nowak and Bangham (14) obtained a mathematical interpretation of viral decay data presented in HIV patients treated with anti-HIV drugs. Since then, modeling HIV infection has been a very active research topic over the past decades. Most of these modeling works initially aimed to represent the basic relation between the host cells and virus (15–21). In addition, significant efforts were invested to understand HIV disease progression (22–29), viral persistence (30–34), drug resistance (35–39), and optimal cART scheduling (40–46) among many others. Mathematical modeling was also pointed out as a tool to assess the potential of “kick-kill” strategies on long-term outcomes from short-term studies (47).

This short review focuses on discussing briefly the uncharted territory of HIV eradication as well as the most recent mathematical modeling contributions aiming to shed light on major clinical implications toward an HIV cure, see Table 1.

**Table 1:** Mathematical models discussing an HIV cure.

Aim	Source	Modeling approach	Prediction
Posttreatment control	Hill et al. (48)	Branching Process	A 5.8-log reduction in the reservoir size is necessary to prevent viral rebound for 95% of cases with cART interruption. Approximately 2,000-fold reduction in the reservoir size is required for 1 year cART interruption without viral rebound.
Posttreatment control	Pinkevych et al. (49)	Exponential model	Viral replication is initiated on average every 6 days. Only 50–70-fold reduction in the reservoir size is required for 1 year cART interruption without viral rebound.
Posttreatment control	Conway et al. (50)	ODEs	Viral rebound depends on the size of the latent reservoir and CTL strength.
Vorinostat treatment	Ke et al. (51)	ODEs	A multistage delay activation model can recapitulate the UsRNA changes induced by vorinostat. Vorinostat may not induce killing of transcriptionally activated cells leading to a minimal reservoir reduction.
Romidepsin treatment	Policicchio et al. (52)	ODEs	The slopes of plasma viral load increase after romidepsin treatment are related to the intensification in viral replication attributed to romidepsin. The estimated slope was 0.418 log_10_/day.
Relation between HIV reactivation and reservoir reduction	Petravic et al. (53)	ODEs	The half-life of cells reactivated with panobinostat is >1 month while with romidepsin is 2 days. The increase in reactivation rate baseline by panobinostat is approximately 8% and around 2.5-fold increase for romidepsin.
Immunization	Luo et al. (54)	Markov process	Competitive exclusion by autologous antibodies may prevent the appearance of broadly neutralizing antibodies.
Immunization	Wang et al. (55)	Agent-based model	Sequential immunization with different antigens is better than a cocktail for induction of cross-reactive antibodies. Antigen variants can impair antibody maturation.

## 2 “Shrinking” the Reservoirs

A very debatable question in HIV research has been if the existence of an HIV latent cellular reservoir is maintained by long-lived resting memory CD4+ T cells or through residual virus replication that replenishes the HIV reservoir (2, 56). Till now, latently infected resting memory CD4+ T cells are the only cell type in which it has been clearly demonstrated that replication-competent virus can persist for several years in patients (4, 57–59).

Memory CD4+ T cells represent the largest lymphocyte population in the adult human body and play critical roles in maintaining a life-long immune defense against specific pathogens (60). Reservoir maintenance is disputable, it has been mainly attributed to the replenishment of the pool, presumably by homeostatic or antigen-driven clonal proliferation and *de novo* infection of memory CD4+ T cells, ensuring the continuous replenishment of the HIV reservoir (57). Recent works from different labs (4, 57–59) revealed a progressive reduction of the size of the blood latent reservoir around a core of less-differentiated memory subsets (central memory and stem cell-like memory CD4+ T cells). These works indicated an extreme stability of different sub-reservoirs, the size of which is directly related to cumulative plasma virus exposure before the onset of cART (58, 59), stressing the importance of early initiation of effective cART. Nevertheless, very recent studies demonstrated that the viral reservoir is seeded rapidly after SIV infection of rhesus monkeys, even before detectable viremia (61). Therefore, the multifactorial mechanisms of HIV reservoirs and their establishment according to the time of optimal cART are still a matter of debate.

The VISCONTI study (9) dissected for the first time that the initiation of cART at very early stages of infection could decrease the size of the HIV reservoirs. In this study, cART was provided for 3 years after primary infection (PHI) to 14 patients and then interrupted. This study revealed that the 14 patients presented a sustained control for a median of 7 years named as posttreatment controllers (PTCs), implying that perhaps the nature of the viral reservoir (levels of TCM cells) could play an important role in controlling the infection in the absence of cART. Note that PTCs are not the only ones that can control HIV infection, a small group of individuals identified in 2005 showed the ability to control HIV infection in the absence of cART named as “Elite controllers (ECs)” (62). Although both ECs and PTCs can control the HIV infection, ECs can naturally maintain undetectable viral loads mainly attributed to a measurably stronger CTL response than non-controllers. Note that ECs undetectable viral loads are not only due to strong CTL but also other cell types, HLA type, and cytokines and chemokines (63).

Although the VISCONTI study revealed crucial information for a successful therapeutic strategy inducing viral remission, it left open several questions, in particular, how can we increase the probability of HIV-infected patients becoming PTC? The answer is not intuitive, in this direction, Hill et al. (48) proposed a mathematical model based on a two-type branching process assuming only four types of events: a latently infected cell can either activate or die, an actively infected cell can either die or produce a collection of virions that results in the infection of other cells. The model provided a relevant prediction that it might not be necessary to deplete completely the reservoir pool to prevent viral rebound, representing a PTC. The reasoning behind these predictions is that the high variability in viral progeny generated from actively infected cells increases the probability that the progeny of an activated provirus will go extinct because of stochastic diffusion. Hill et al. (48) results suggested that in order to achieve the goal of eradication for 95% of patients, a 5.8 logs reduction in the reservoir size may be necessary. Alternatively, to reach 1 year average without rebound, approximately 2,000-fold reduction would be required (48).

Controverting Hill et al. (48) predictions, Pinkevych et al. (49) combined data from four independent clinical cohorts of patients with cART interruption together using a simple model with exponential phase with a “shoulder” that represents the time for drug “washout” and viral growth. Pinkevych et al. (49) estimated that viral replication is initiated on average once every 6 days approximately, which is about 24 times lower than previous estimations. Furthermore, the model indicated that a modest 50–70-fold reduction of the reservoir would be required for 1 year without viral rebound after cART interruption. Six months later, Hill et al. (64) questioned the estimation approach by Pinkevych et al. (49). Mathematical models including modest interpersonal variations in Ref. (64) were also able to explain the observed variation in rebound times, rejecting the simplifying assumption of homogeneity in Ref. (49). In response to the critic of Hill et al. (64), Pinkevych et al. (65) derived an analytical approximation incorporating multiple reactivation events and consequently fitted to four datasets. Overall, fitting results in Ref. (65) indicated similar results to their original work (49). Furthermore, Pinkevych et al. (65) argued back to the work in Hill et al. (48) for using reactivation rates from previous publications that were not based on data on reactivation from latency after treatment interruption.

In a separate modeling lab, Conway et al. (50) tested the immune system response led by cytotoxic T cell (CTL) would be sufficient to control the infection due to the rate of new productively infected cells is small. To this end, using ordinary differential equations (ODEs), numerical simulations in Ref. (50) pointed out that for very strong CTL responses, HIV infection would be controlled in a similar fashion as with the ECs. Moreover, within the first 6 months of cART interruption, the model represented qualitatively similar the viral rebound to those reported in some individuals in the VISCONTI study. Interestingly, the model analysis reveals that not only a very low latent reservoir size is necessary to guarantee PTCs but also the CTL strength. A shortcoming of the Conway et al. model is the missing quantitative information to precise CD8+ T response leading to a subjective selection of the model and parameters.

## 3 “Kicking” the Latent Reservoirs

The central dogma for an HIV cure is to reverse latency of resting CD4+ T cells that harbor replication competent proviruses (30), but at the same time, a major challenge in “purging” treatment strategies is the reduction of the virus without causing global T cell activation (66). The ability to induce HIV viremia or at least cell surface expression of viral proteins and presentation of viral antigens is a fundamental requirement for enabling the immune-mediated killing of latently infected cells and, thus, defines the key goal of latency-reversing agents (LRAs) in eradication strategies (11). These agents include histone methyltransferase inhibitors and histone deacetylases (HDAC) inhibitors (67). The potential to activate HIV production from latently infected cell lines and resting CD4+ T cells from HIV-infected patients on suppressive cART is under large debate last 5 years (11, 68). Thus far, multiple HDAC inhibitors can potently activate viral production *in vitro*, however, results of initial clinical trials in HIV-infected patients are just very recently published (10, 69, 70).

Essential steps in the life cycle of HIV within host cells, cell-associated HIV RNA markers, have been identified and currently used in several clinical trials as a surrogate to measure the degree of HIV persistence (71). Among HIV RNA markers, unspliced RNA (UsRNA) also referred as cell-associated unspliced (CA-US) is more easy to detect and thus several studies have linked to virus persistence (71).

For vorinostat, a promising LRA, 20 HIV-infected individuals on suppressive cART were treated with 400 mg of vorinostat for 14 days and then followed by 70 days (69). Although highly variable outcomes among the participants, vorinostat induced a significant and sustained increase UsRNA. Ke et al. (51) proposed three different models based on ODEs to further understand how latently infected cells respond dynamically to vorinostat: a direct activation model, a delay activation model, and a multistage delayed model. The model analysis in Ref. (51) revealed that a multistage delayed activation model could recapitulate the short-term and the long-term changes induced by vorinostat in UsRNA in most of the participants. This can be interpreted as latently infected cells may need to go through several stages before becoming sustainably activated. Clinically relevant for HIV persistence, parameter estimates by Ke et al. (51) evoked the idea that vorinostat treatment may not induce killing of transcriptionally activated cells leading to a minimal or absent reduction in reservoir size.

Another important LRA is romidepsin, Søgaard et al. (70) reported in a small clinical trial of six HIV-infected individuals who received romidepsin once weekly for 3 weeks while keeping cART. In contrast to vorinostat that did not induce plasma HIV RNA, romidepsin treatment promoted in five patients an increase of plasma HIV RNA to detectable levels ranging from 46 to 103 copies/ml. However, romidepsin and vorinostat did not alter the size of the HIV reservoir (72). To demonstrate that romidepsin may successfully activate the latent reservoir, Policicchio et al. (52) developed a non-human primate (NH) model to capture the characteristics of PTCs. Unexpectedly, stopping cART 7 days after romidepsin administration showed that viral rebound occurred as early as 3 days after cART interruption. Note that the average time of viral rebounds in humans is approximately 8 weeks (73). Employing a simple mathematical model of viral production, Policicchio et al. (52) indicated that the slopes of plasma viral load increase after romidepsin treatment are related to the intensification of viral replication attributed to romidepsin. Fitting results showed that the estimated slope was 0.418 log_10_/day.

Thus far, in clinical trials, HDACs have demonstrated an increase of UsRNA in total but a minimal reduction in reservoir size. Based on simple mathematical models assuming “direct activation,” Petravic et al. (53) suggested that several mechanisms such as maintenance and clearance of the reservoirs as well as other mechanisms may significantly impact the relationship between HIV reactivation and the reduction of latently infected cells. In particular, Petravic et al. (53) considered the impact of panobinostat and romidepsin, both drugs revealed 3–4 increased of CA-RNA in clinical trials. On one hand, cells reactivated with panobinostat have a long life span (half-life >1 month) suggesting a modest increase in reactivation rate (approximately 8%). On the other hand, cells activated with romidepsin have a short life span (2 days), implying that HIV reactivation rate may have doubled with romidepsin (53).

Overall, it is envisaged that additional interventions will be needed to eliminate efficiently latently infected cells (69). It is, therefore, very likely that HDACs will form part of a multipronged strategy (74). Consequently, mathematical models merging dynamics from different HDAC inhibitors may help to propose “kick” strategies to eliminate latently infected cells to achieve the ultimate goal of HIV eradication.

## 4 “Killing” the Activated Reservoir

Purging strategies reached a new level of complexity due to recently published works addressing the frequency of CTL escape mutations in archived proviruses, indicating an unexpected and exceptionally dynamic nature of the latent reservoir (75). Although cART is started early, the vast majority of latent viruses carry CTL mutations that render infected cells unrecognizable by CTLs directed at common epitopes. While the non-protective responses may not be harmful *per se*, they may dominate and suppress the true protective ones. Thus, it is critical to (re)focus T-cell responses on the protective, biologically conserved epitopes of the HIV-1-proteome by effective vaccination (76). However, the biggest obstacle for vaccine development is the HIV-1 variability and escape from mounted responses. T-cell strategies focus on vaccine-elicited responses on the most conserved regions of the HIV-1 proteome are very promising, due to these are common to most variants and cause replicative fitness loss if mutated (76, 77). In this context, the best “kill” strategy could be based on innovative vaccines aiming to induce CD8+ T cell responses in conserved regions of the HIV-1 proteome.

To the best knowledge of the author, there is not any mathematical work to evaluate vaccines aiming to induce CD8+ T cell responses. Till now, mathematical models have focused on incorporating affinity antibody maturation (54, 55). Using agent-based simulations of the Germinal Center (GC) reaction, simulation results from Wang et al. (55) suggested that the induction of cross-reactive antibodies occurs with low probability because of conflicting forces by different antigens, ultimately frustrating affinity maturation. Wang et al. (55) provided a critical prediction that sequential immunization with different antigens would be preferred over a cocktail for induction of cross-reactive antibodies. In a similar vein, Luo et al. (54) proposed a Markov process model to simulate coevolving multi-type virus and antibodies populations. Simulations results provided also the hint that competitive exclusion by autologous antibodies could avoid the appearance of broadly neutralizing antibodies.

## 5 A Roadmap for HIV Modeling

HIV modeling is on uncharted territory. Modeling “kill” strategies aiming to induce CD8+ T cell responses in cooperation with a combination of HDAC inhibitors has the potential to advance understanding toward HIV eradication. There are at the moment several clinical trials based on “kick-kill” therapies such as the RIVER study (78), for which a long-term follow-up out to 5 years is envisaged. Furthermore, several mechanisms may be underestimated in mathematical modeling research. Recent experimental evidence revealed that clonal proliferation of infected cell may play a central role maintaining the reservoirs (79). On the other hand, viral control may not be only associated with restoration of CD8+ T cells (80). Mathematical models presented till now assume only T cell responses as a main component. However, further modeling efforts including host factors and immune responses responsible for the HIV elite status may uncover clues for the design of therapeutic vaccines and functional cures (63). Ultimately, mathematical models of HIV compartments (e.g., different places where the virus is present) and sanctuaries (e.g., limited penetration of drugs that maintains persistent replication) are needed to weight HIV persistence.

Above all, the difference in opinion of modeling approaches between Pinkevych et al. and Hill et al. points out that there is a great need to unify the efforts in modeling practices such as develop good practice guidelines for reporting parameter fitting results. Although assuming there exists a model that represents properly the problem at hand, model fitting to experimental data is subject to a large number of factors that can distort parameter estimates (81). Efforts in dealing with errors in parameter estimation shall be well documented in next mathematical models to strengthen and support further development toward HIV eradication.

## Author Contributions

EAHV conceived and wrote the manuscript.

## Conflict of Interest Statement

The author declares that the research was conducted in the absence of any commercial or financial relationships that could be construed as a potential conflict of interest.
